# Supplementary material for: Comparative Volatilomic Profile of Three Finger Lime (Citrus australasica) Cultivars Based on Chemometrics Analysis of HS-SPME/GC–MS Data
Source: Molecules. 2022 Nov 14;27(22):7846. doi: 10.3390/molecules27227846 (PMC9697472; doi:10.3390/molecules27227846)
Supplement: Supplementary file 1 [file molecules-27-07846-s001.zip › molecules-1962707-supplementary.pdf]

SUPPLEMENTARY MATERIALS

Comparative volatilomic profile of three finger lime (*Citrus australasica*) cultivars based on chemometrics analysis of HS-SPME/GC-MS data

Rosaria Cozzolino [1], José S. Câmara [2,3*], Livia Malorni[1], Giuseppe Amato [1], Ciro Cannavacciuolo [4], Milena Masullo[4] and Piacente Sonia[4*]

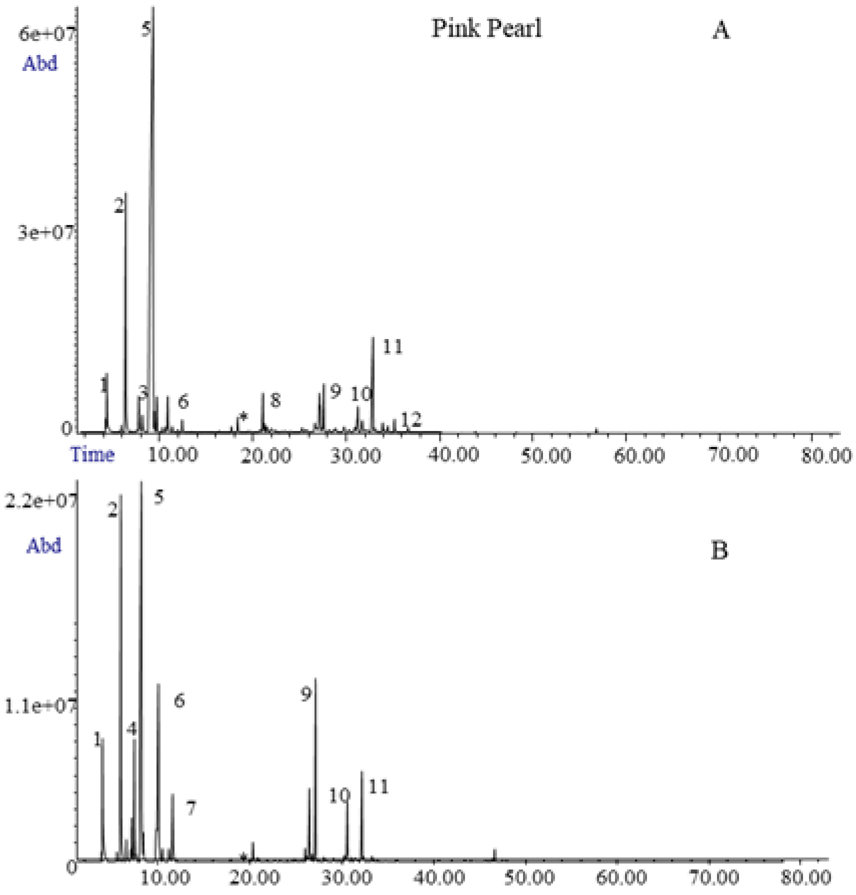

Figure S1. Representative TICs of the peel (A) and the juice (B) of Pink Pearl cv. (1) α-Thujene, (2) Sabinene; (3) β-Myrcene; (4) α-Terpinene; (5) Limonene; (6) γ- Terpinene; (7) α-Terpinolene; (8) δ-Elemene; (9) 4-Terpineol; (10) Ledene; (11) Bicyclogermacrene; (12) β-Citronellol; *IS

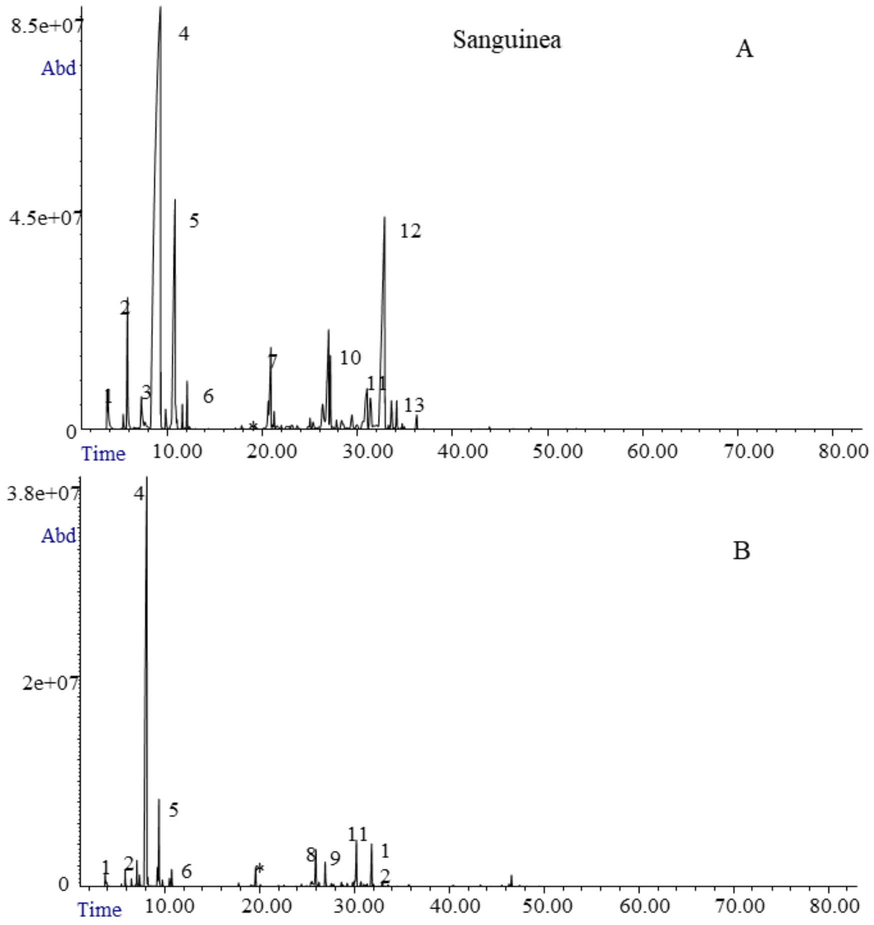

Figure S2. Representative TICs of the peel (A) and the juice (B) of Sanguinea cv. (1) α-Pinene; (2) Sabinene; (3) β-Myrcene; (4) Limonene; (5) γ- Terpinene; (6) α-Terpinolene; (7) δ-Elemene; (8) Aromadendrene; (9) Isoledene; (10) 4-Terpineol; (11) Ledene; (12) Bicyclogermacrene; (13) α-Farnesene; *IS

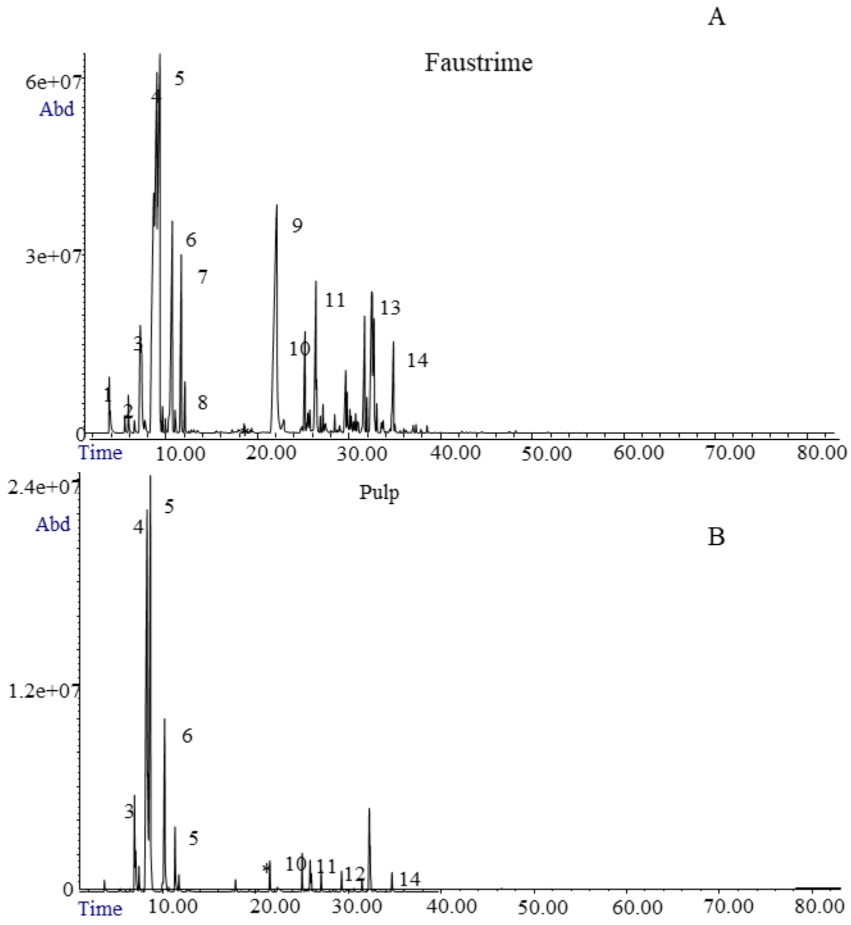

Figure S3. Representative TICs of the peel (A) and the juice (B) of Faustrime cv. (1) α-Pinene; (2) Sabinene; (3): α-Phellandrene; (4) Limonene; (5) β-Phellandrene; (6) γ- Terpinene; (7) p-Cymene; (8) α-Terpinolene; (9) Citronellal; (10) Linalool; (11) α-Bergamotene; (12) α-Caryophillene; (13) β-Bisabolene; (14) β-Citronellol; *IS

Table S1. Volatile metabolites detected in the three cv of *Citrus australasica* Mill. and their identification codes

Metabolite	Code	[a]RI/[b]RI	[c]ID	Metabolite	Code	[a]RI/[b]RI	[c]ID
**Esters**							
Ethyl acetate	E1	871/871	RI/MS/S	δ-3-Carene	MH6	1135/1135	RI/MS/S
cis-3-Hexen-1-ol acetate	E2	1323/1323	RI/MS	α-Phellandrene	MH7	1160/1160	RI/MS
Hexen-1-ol propionate	E3	1387/1390	RI/MS	β-Myrcene	MH8	1169/1169	RI/MS/S
Hexyl butyrate	E4	1419/1419	RI/MS/S	α-Terpinene	MH9	1184/1184	RI/MS/S
**Aldehydes**				D-Limonene	MH10	1227/1226	RI/MS/S
2-Butenal	Ald1	1047/1047	RI/MS	β-Phellandrene	MH11	1228/1228	RI/MS/S
Hexanal	Ald2	1084/1084	RI/MS/S	cis-β-Ocimene	MH12	1248/1248	RI/MS/S
cis-3-Hexenal	Ald3	1152/1152	RI/MS	γ-Terpinene	MH13	1253/1253	RI/MS/S
2-Hexenal	Ald4	1238/1238	RI/MS/S	trans-β-Ocimene	MH14	1261/1261	RI/MS/S
Octanal	Ald5	1297/1297	RI/MS/S	p-Cymene	MH15	1274/1274	RI/MS/S
2-Heptenal	Ald6	1333/1333	RI/MS	α-Terpinolene	MH16	1284/1284	RI/MS/S
Nonanal	Ald7	1398/1398	RI/MS/S	Allocimene	MH17	1377/1377	RI/MS/S
**Alcohols**				cis-Sabinene hydrate	MH18	1469/1469	RI/MS
2-Penten-1-ol	Al1	1332/1333	RI/MS	trans-Sabinene hydrate	MH19	1548/1546	RI/MS
1-Hexanol	Al2	1364/1364	RI/MS/S	**Oxygenated Monoterpenes**			
3-Hexen-1-ol	Al3	1391/1392	RI/MS/S	*cis*-Limonene oxide	MO1	1439/1430	RI/MS
2-Hexen-1-ol	Al4	1414/1414	RI/MS/S	Citronellal	MO2	1480/1480	RI/MS
Benzenmethanol	Al5	1846/1846	RI/MS/S	Linalool	MO3	1553/1553	RI/MS/S
**Monoterpenes Hydrocarbons**				Isopulegol	MO4	1565/1565	RI/MS
α-Pinene	MH1	1015/1015	RI/MS/S	Terpinen-4-ol	MO5	1595/1595	RI/MS/S
α-Thujene	MH2	1021/1021	RI/MS/S	Carvone	MO6	1722/1722	RI/MS/S
Camphene	MH3	1052/1052	RI/MS/S	β-Citronellol	MO7	1770/1770	RI/MS/S
β-Pinene	MH4	1087/1087	RI/MS/S	*cis*-p-Mentha-1(7),8-dien-2-ol	MO8	1790/1790	RI/MS
Sabinene	MH5	1112/1112	RI/MS/S	*trans*-Carveol	MO9	1833/1836	RI/MS/S
				cis-Carveol	MO10	1863/1846	RI/MS/S
				Methyleugenol	MO11	2012/2012	RI/MS
**Sesquiterpenes**							
α-Cubebene	Sesq1	1449/1449	RI/MS	Cadina 1,4 diene	Sesq26	1765/1768	RI/MS
α-Copaene	Sesq2	1452/1454	RI/MS	γ-Muurolene	Sesq27	1735/1731	RI/MS

δ-Elemene	Sesq3	1463/1468	RI/MS	Germacrene B	Sesq28	1805/1805	RI/MS
Bicycloelemene	Sesq4	1471/1471	RI/MS	Calamenene	Sesq29	1811/1812	RI/MS
β-Bourbonene	Sesq5	1504/1510	RI/MS	α-Calacorene	Sesq30	1897/1901	RI/MS
α-Gurjunene	Sesq6	1514/1515	RI/MS	Epiglobulol	Sesq31	2001/2000	RI/MS
Aristolene	Sesq7	1544/1552	RI/MS	Nerolidol	Sesq32	2045/2045	RI/MS/S
α-Bergamotene	Sesq8	1562/1542	RI/MS	Sphatulenol	Sesq33	2114/2129	RI/MS
β-Elemene	Sesq9	1568/1593	RI/MS	**Others**			
Calarene	Sesq10	1571/1544	RI/MS	2-Ethyl-furan	O1	945/945	RI/MS/S
Aromadendrene	Sesq11	1620/1622	RI/MS/S	Tridecane	O2	1296/1300	RI/MS/S
Epizonarene	Sesq12	1627/1672	RI/MS	Tetradecene	O3	1426/1428	RI/MS
γ-Gurjunene	Sesq13	1634/1635	RI/MS	Tetradecane	O4	1402/1400	RI/MS/S
Epibicyclosesquiphellandrene	Sesq14	1643/1669	RI/MS	Pentadecane	O5	1503/1500	RI/MS/S
Valencene	Sesq15	1646/1715	RI/MS				
α-Caryophyllene	Sesq16	1648/1663	RI/MS/S				
β-Guaiene	Sesq17	1673/1671	RI/MS				
Ledene	Sesq18	1681/1679	RI/MS				
Germacrene D	Sesq19	1692/1696	RI/MS				
β-Selinene	Sesq20	1699/1698	RI/MS				
α-Selinene	Sesq21	1704/1704	RI/MS				
Bicyclogermacrene	Sesq22	1722/1736	RI/MS				
β-Bisabolene	Sesq23	1724/1723	RI/MS				
δ-Cadinene	Sesq24	1742/1749	RI/MS				
α- Farnesene	Sesq25	1745/1745	RI/MS				

[a]Relative retention indices calculated against n-alkanes (C_8-C_{20}) on HP-Innowax column; [b]Relative retention indices on polar column reported in literature for polar column (www.pherobase.com, www. flavornet.org, www.ChemSpider.com, https://pubchem.ncbi.nlm.nih.gov, webbook.nist.gov).; [c]Identification method as indicated by the following: RI -~~—~~ ~~Kovats~~ Relative retention index on a ~~on~~ HP-Innowax column; MS -~~—~~ NIST and Wiley libraries spectra; S -~~—~~ co-injection with authentic standard compounds on the HP-Innowax column. E: esters; Al: alcohols; Ald: aldehydes; MH: monoterpene hydrocarbons; MO: oxygenate monoterpenes; Sesqu: sesquiterpenes; O: Others.
